# Prevalence of abuse among young children with femur fractures: a systematic review

**DOI:** 10.1186/1471-2431-14-169

**Published:** 2014-07-02

**Authors:** Joanne N Wood, Oludolapo Fakeye, Valerie Mondestin, David M Rubin, Russell Localio, Chris Feudtner

**Affiliations:** 1 Division of General Pediatrics and PolicyLab, The Children’s Hospital of Philadelphia, 3535 Market Street, Floor 15, Philadelphia PA19104, Pennsylvania; 2 Leonard Davis Institute of Health Economics, Colonial Penn Center, 3641 Locust Walk, Philadelphia 19104, Pennsylvania; 3 Department of Pediatrics, Perelman School of Medicine at the University of Pennsylvania, Philadelphia 19104, Pennsylvania; 4 Department of Biostatistics and Epidemiology, Perelman School of Medicine at the University of Pennsylvania, Philadelphia 19104, Pennsylvania

**Keywords:** Child abuse, Child maltreatment, Femur fracture, Accident, Trauma

## Abstract

**Background:**

Clinical factors that affect the likelihood of abuse in children with femur fractures have not been well elucidated. Consequently, specifying which children with femur fractures warrant an abuse evaluation is difficult. Therefore the purpose of this study is to estimate the proportion of femur fractures in young children attributable to abuse and to identify demographic, injury and presentation characteristics that affect the probability that femur fractures are secondary to abuse.

**Methods:**

We conducted a systematic review of published articles written in English between January 1990 and July 2013 on femur fracture etiology in children less than or equal to 5 years old based on searches in PubMed/MEDLINE and CINAHL databases. Data extraction was based on pre-defined data elements and included study quality indicators. A meta-analysis was not performed due to study population heterogeneity.

**Results:**

Across the 24 studies reviewed, there were a total of 10,717 children less than or equal to 60 months old with femur fractures. Among children less than 12 months old with all types of femur fractures, investigators found abuse rates ranging from 16.7% to 35.2%. Among children 12 months old or greater with femur fractures, abuse rates were lower: from 1.5% - 6.0%. In multiple studies, age less than 12 months, non-ambulatory status, a suspicious history, and the presence of additional injuries were associated with findings of abuse. Diaphyseal fractures were associated with a lower abuse incidence in multiple studies. Fracture side and spiral fracture type, however, were not associated with abuse.

**Conclusions:**

Studies commonly find a high proportion of abuse among children less than 12 months old with femur fractures. The reported trauma history, physical examination findings and radiologic results must be examined for characteristics that increase or decrease the likelihood of abuse determination.

## Background

Femur fractures are the most common orthopedic injury for which children are hospitalized in the United States [1,2]. Although the majority of childhood femur fractures result from accidental trauma, abuse is also a common cause of these fractures, especially in children less than 1 year old. Thus, medical providers caring for children with femur fractures should recognize and evaluate children who might be abuse victims. Abuse evaluation and diagnosis rates among children with femur fractures have, however, been noted to vary among hospitals and providers [3-6]. Furthermore, studies have shown that failing to recognize and evaluate for abuse in young children with fractures can result in children suffering complications from additional undiagnosed injuries as well as ongoing abuse [7].

Although femur fractures have been associated, in general, with a high abuse risk in young children, [8-10] the prevalence of abuse in children with different types of femur fractures has not been established. Moreover, other clinical features that increase or decrease the likelihood of abuse determination in children with femur fractures have not been well elucidated. This uncertainty regarding which children with femur fractures might have been abused may contribute to variation in care and missed opportunities to diagnose abuse in the pediatric population.

Given these uncertainties, we systematically reviewed published studies in order to: 1) provide estimates of abuse prevalence among children ≤5 years old with femur fractures and 2) describe the association of specific clinical features with likelihood of abuse determination. Recognizing that the vast majority of abusive fractures occur in the infants and young toddlers, we included children up to age 5 years in our review as abusive fractures have been occasionally reported in preschool age children [10-13]. Due to the heterogeneity of study populations, we did not perform a meta-analysis. Instead, we present the proportions of children diagnosed with abuse in each study as well as details of the study population, in order to provide a richer understanding of the prevalence of abuse in different sub-populations of children with femur fractures.

## Methods

### Search strategy

**Figure 1:**
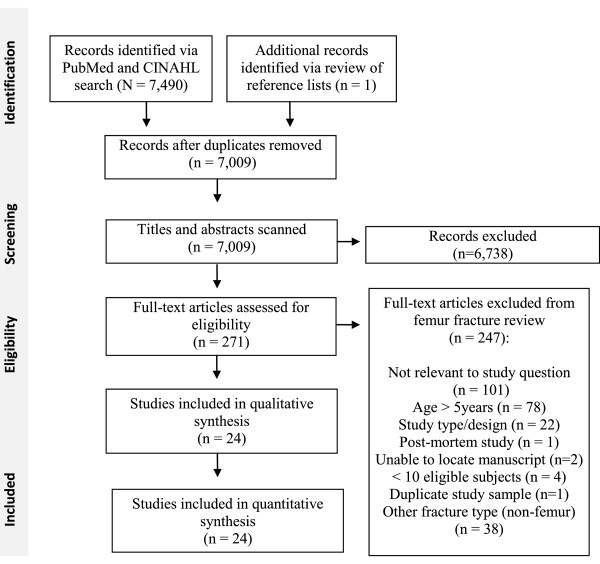
Processes of study identification, screening, eligibility assessment and inclusion.

A systematic review of the literature on abuse in children with fractures was performed using a pre-specified protocol with inclusion criteria (available upon request). This paper covers the subset of articles specific to femur fractures (Figure 1). We performed searches for studies published in English between January 1990 and July 2013 in the PubMed/MEDLINE and CINAHL databases using the search terms listed in Additional file 1. We included terms related to both abuse and accidental trauma to avoid bias toward studies focused exclusively on abuse. Studies were also identified by iteratively reviewing reference lists of articles identified during the search.

### Study selection

Randomized controlled trials (RCT), prospective non-RCT studies, and retrospective data analyses were included; surveys, reviews, editorials, case series and textbooks were excluded. Studies were included if subjects were ≤5 years old or if the data for the subset of children ≤5 years old could be extracted. Studies including fewer than 10 children ≤ 5 years old with femur fractures were excluded, as were animal and post-mortem studies. Methodologically weak studies due to significant bias in selection of subjects, such as studies including only cases seen by the investigator for medical-legal review or studies including only the subset of patients who were eligible for a specific treatment modality, were excluded. Titles and abstracts of studies were screened by one of four reviewers (JW, OF, Maria Fatima de Reyes, VM), and non-relevant studies were eliminated (Figure 1). Full manuscripts for relevant studies were assessed for eligibility by two reviewers (JW and OF or VM) in an unblinded standardized manner, with disagreements resolved by consensus.

### Data extraction, assessment of methodological quality, and analysis

Two reviewers (JW, OF) independently extracted the following information from the studies using a standardized form: 1) study population characteristics (ages, type(s) of fractures, study location and dates), 2) inclusion and exclusion criteria for subjects, 3) potential biases, and 4) number of fractures attributed to abuse and accidental mechanisms in overall study population as well as within study subpopulations. The reviewers assessed the level of the evidence presented in each study using the 2011 Oxford Centre for Evidence-Based Medicine (CEBM) Levels of Evidence table (Table 1). Per the CEBM Evidence Levels, articles providing the strongest evidence are likely to be assessed as a level 1, and those providing the weakest evidence are likely to be assessed as a level 5 [14,15]. The CEBM Evidence Levels for “How common is the problem” were applied to studies of the prevalence of abuse in children with femur fractures. The CEBM Evidence Levels for diagnosis studies were adapted for assessing studies examining the association of specific clinical features with likelihood of abuse determination. Studies providing information on both questions were assigned a single score based on the question for which they had the weakest level of evidence. The level of evidence assigned to a study is specific to the level of evidence for answering the questions posed in this review and may not be reflective of the overall quality of the study. For example, the level of evidence was downgraded for studies with small numbersof children with femur fractures although the studies may have had appropriate methods and large study populations that included children with other types of fractures. Similarly, studies were classified as non-current and received a lower level if any of the data was from prior to 2000. Finally, the CEBM Evidence Levels for prevalence studies include assessment of whether the study population is local or not, but we chose to eliminate this factor as we were not attempting to estimate the prevalence of abuse in a particular location. Instead we provide the location of each study and leave it to the reader to determine the applicability of the study data to their population of interest. For each study, the reviewers also rated the methodology used to determine that an injury was due to abuse using a scale adapted from Maguire et al. [16] which assigned the highest rank (1) to studies requiring that abuse be either confirmed at a child abuse case conference or civil or criminal court proceedings, or admitted by a perpetrator, or witnessed. The lowest rank (5) was accorded to studies providing no stated criteria for categorizing cases as suspected abuse (Table 2). Reviewers resolved disagreements through discussion and consensus.

**Table 1:** Study methodology rating scales; levels of evidence scale*

**Question**	**How common is physical abuse?**	**Is this factor associated with risk of physical abuse?**
Level 1	Current random sample surveys (or censuses)	Systematic review of cross sectional studies with consistently applied reference standard and blinding
Level 2	Systematic review of surveys that allow matching to local circumstances	Individual cross sectional studies with consistently applied reference standard and blinding
Level 3	Non-random or non-current sample	Non-consecutive studies, or studies without consistently applied reference standards
Level 4	Case-series	Case–control studies, or “poor or non-independent reference standard
Level 5	n/a	Mechanism-based reasoning

*Adapted from the 2011 Oxford CEBM Evidence Levels of Evidence table [14]. Level may be graded down on the basis of study quality, imprecision, indirectness, or because the absolute effect size is very small. Current was defined as data from 2000 or later.

**Table 2:** Study methodology rating scales; abuse determination methodology rating scale*

	
1	Abuse confirmed at case conference or family, civil, or criminal court proceedings; admitted by perpetrator; or witnessed abuse
2	Abuse confirmed by stated criteria including multidisciplinary assessment
3a	Abuse defined using specific stated case based criteria
3b	Abuse including cases of likely or probable abuse defined by specific stated case based criteria
4	Abuse stated but no supporting detail given as to how a determination of abuse was made^#^
5	Suspected abuse

*Adapted from a scale with 5 levels developed by Maguire et al. (2005). [16] For studies using specific stated case based criteria to make a determination of abuse (rating 3), we distinguished studies that included only definite abuse cases (3a) from those that also included likely or probable abuse cases (3b). Assessment by multidisciplinary hospital-based child protection team as part of routine clinical care did not qualify as multidisciplinary assessment. ^#^Level 4 includes studies relying on ICD-9 and E-codes for identifying abuse cases in administrative data sets and studies relying on diagnoses of abuse made by clinical teams without providing specific criteria by which these diagnoses were made.

The abuse prevalence for each study was calculated with 95% confidence intervals (CIs) from the data reported in cohort and cross-sectional studies. The study-specific sensitivity, specificity, positive likelihood and negative likelihood ratios with 95% CIs of different clinical characteristics for abuse were also computed. Formulae described by Simel et al. [17] were applied to calculate 95% CIs for likelihood ratios. All other analyses were performed using Stata 12 (StataCorp, College Station, TX). The Preferred Reporting Items for Systematic Reviews and Meta-Analyses (PRISMA) checklist was utilized in reporting the results of the systematic review (Additional file 2).

## Results

### Description of studies

The comprehensive literature search identified 7,009 non-duplicate citations on all fractures, of which 271 were deemed relevant. Twenty-four studies on femur fractures met inclusion criteria (Figure 1). [2,4-6,10,18-36] A total of 10,717 children of ages 0–5 with femur fractures were examined in the 24 studies. Two studies using a national database [10,19] received a study methodology quality rating of L1, 1 study [31] received a rating of L2, 3 studies [2,20,24] received a rating of L5, and the remainder of studies received ratings of L3 or L4 (Table 3) [4-6,18,21-23,25-30,32-36]. Three studies requiring that abuse be confirmed by case conference or court proceedings following a child protective services (CPS) investigation received a rating of 1 for the abuse determination methods [18,22,26]. Ten studies reported specific clinical criteria applied in diagnosing abuse (rating 2 or 3) [4,20,21,24,25,29-31,33,36]. Eleven studies, including 8 studies relying on administrative data, received abuse determination ratings of 4 or 5 [2,5,6,10,19,23,27,28,32,34,35].

**Table 3:** Summary of study characteristics

**Study (1**^**st **^**author, year)**	**Study Location/Source**	**Dates**	**Age (mo)**	**Inclusion criteria**	**Exclusions**	**Study Methods Ranking**^**a**^	***n***
Dalton, 1990 [4]	3 Michigan Hospitals, USA	1979-1983	<36	Femur fracture all types	Additional injuries^b^	L4 / 3a	138
Thomas, 1991 [20]	Yale-New Haven Hospital, USA	1997-1984	<36	Femur fracture all types	Pathologic fracture	L5 / 3b	25^c^
Kowal-Vern, 1992 [21]	Loyola University Medical Center, USA	1984-1989	<36	Femur fracture all types	None	L4 / 3a	14^c^
Blakemore, 1996 [22]	C.S. Mott Children’s Hospital, USA	1979-1993	12-71	Isolated femur diaphyseal fracture	Pathologic fractures, MVC related fractures, additional injuries^b^	L4 / 1	42
Hinton, 1999 [23]	Hospital Discharge Database of the Maryland Health Services Cost Review Commission, USA	1995-1996	<24	Femur diaphyseal fractures	Non-acute fracture, multiple admissions	L3 / 4	73^c^
Rex, 2000 [24]	Manchester Children’s Hospitals, UK	1992-1996	<60	Femur fracture all types, definite abuse or accident	Unclear etiology, non-acute fractures	L5 / 3a	33^c^
Scherl, 2000 [25]	The University of Chicago Children’s Hospital & King’s County Hospital, USA	1986-1996	<72	Closed diaphyseal femur fracture	Non-diaphyseal fractures, open fracture, pathologic fracture	L3 / 2	207
Schwend, 2000 [26]	Children’s Hospital of Buffalo, New York, USA	1993-1997	<48	Diaphyseal femur fracture	Pathologic fracture, additional injuries^b^	L4 / 1,3b	139
Banaszkiewicz, 2002 [36]	Royal Aberdeen Children’s Hospital, UK	1995-1999	<12	Femur fracture all types	None	L4 / 3b	12^c^
Jeerathanyasakun, 2003 [33]	Queen Sirikit National Institute of Child Health, Thailand	1996-2001	<60	Diaphyseal femur fracture	Non-diaphyseal fracture, distal greenstick fracture, pathologic fracture	L4 / 3b	39
Coffey, 2005 [27]	Children’s Hospital, Columbus, OH, USA	1998-2002	< 18	Femur fracture all types	None	L4 / 5	41^c^
Pierce, 2005 [31]	Children’s Hospital of Pittsburgh, PA, USA	1999-2002	≤36	Femur fracture all types, reported history of stair fall	Reported history other than stair fall	L2 / 3a	29
Rewers, 2005 [35]	Colorado Trauma Registry, USA	1998-2001	<36	Femur fracture all types	Pathologic fracture, non-Colorado resident, repeat admission for complication	L3 / 4	332 ^c^
Loder, 2006 [19]	National (*Kids’ Inpatient Database,* USA)	2000	< 24	Femur fracture all types	None	L1 /4	1,076^c^
Arkader, 2007 [28]	Two Level I pediatric centers, USA	1995-2005	≤ 12	Complete distal metaphyseal femur fracture	Incomplete metaphyseal and epiphyseal fractures	L3 / 4, 5	20
Trokel, 2006 [5]	National (*Kids’ Inpatient Database,* USA)	1997	<12	Femur fracture all types, admitted through ED	MVC, gunshot, or stabbing related fracture; no external cause of injury code	L3 / 4	426^c^
Leventhal, 2007 [29]	Yale-New Haven Children’s Hospital, CT, USA	1979-1983 1991–1994 1999-2002	<36	Femur fracture all types	Pathologic fracture	L4 / 3b	81^c^
Hui, 2008 [30]	Alberta Children’s Hospital, Canada	1994-2005	<36	Femur fracture all types	Pathologic fracture	L4 / 3b	127
Leventhal, 2008 [10]	National (*Kids’ Inpatient Database,* USA)	2003	<36	Femur fracture all types	None	L1 / 4	4,026^c^
Baldwin, 2011 [2]	The Children’s Hospital of Philadelphia, PA, USA	1998+	<48	Femur fracture all types	Pathologic fracture, cause of fracture not clearly determined	L5 / 4	209
Heideken, 2011 [34]	Swedish National Hospital Discharge Registry, Sweden	1987-2005	<12	Diaphyseal femur fracture	Non-diaphyseal fracture, pathologic or birth fracture, multiple femur fractures	L3 /4	313^c^
Shrader, 2011 [32]	Phoenix Children’s Hospital, AZ, USA	2003-2008	<60	Diaphyseal femur fracture	Pathologic fractures, non-diaphyseal fractures	L3 / 4	137
Wood, 2012 [6]	Pediatric Health Information System Database (40 pediatric hospitals), USA	1999-2009	<12	Femur fracture all types	MVC, birth, or neoplasm related fractures	L3 / 4	2,975^c^
Capra, 2013 [18]	The Hospital for Sick Children, Toronto, Canada	1995-2004	12-59	Femur fracture all types	Non ambulatory children, pathological fractures	L3 /1	203

^a^Presents overall study methodology ranking (L1-L5) and abuse determination methodology ranking (1–5). See Table 1 for description of the ranking scales. Some studies utilized multiple different methods to define cases of abuse or suspected abuse and therefore received more than one ranking.

^b^Patients with other injuries (in addition to the femur fracture) were excluded from these studies but the exact definition of additional injuries varied.

^c^The data presented are for the relevant subset of a larger study population which may have included children with other types of injuries, other ages, and/or from other time periods.

The inclusion and exclusion criteria applied in subject selection varied among the 24 studies, with differences in the types of femur fractures and possible etiologies considered. Only 5 studies included children with any femur fracture type from any etiology [10,19,21,27,36]. Thirteen studies excluded children with pathologic fractures and/or children with a clear accidental etiology such as motor vehicle crash (MVC) [2,5,6,18,20,22,26,30,32-35]. Eight included children with specific types of fractures or specific reported trauma histories [22,23,25,26,28,31-34]. Three studies excluded children with additional injuries, [4,22,26] potentially biasing the abuse prevalence lower.

### Abuse prevalence in young children with femur fractures: all types

**Figure 2:**
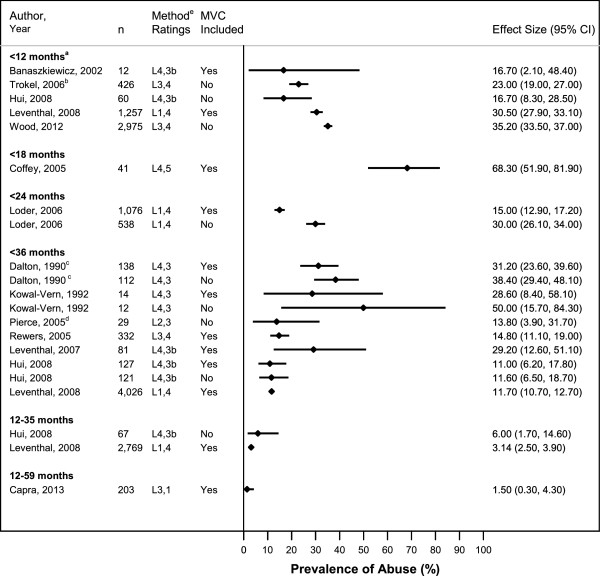
**Probability of Abuse in Children with a Femur Fracture, All Types.** Proportion of cases with abusive femur fractures in included studies, by subject age criteria of inclusion. ^a^Upper age limit was 11 months in Leventhal [10] & Wood [6] and 12 months in Hui [30]. ^b^Data for only the subset of children admitted to children’s hospitals or to general hospitals without children’s hospitals could be extracted. ^c^Study was limited to children with isolated femur fracture and no additional injuries. ^d^Included children with reported history of stair fall ≤36 months old only. ^e^Presents overall study methodology ranking (L1-L5) and abuse determination methodology ranking (1–5).

Among studies including children 0–36 months with all types of femur fractures from any reported etiology, estimated prevalence of abusive fractures ranged from 11.0%-31.2% (Figure 2) [4,10,21,29,30] Exclusion of MVC-related cases increased the range of reported abuse prevalence in the studies to 11.6%-50.0% [4,21,30]. Restricting the population in these studies to children <12 months old resulted in a range of 16.7%-30.5% abuse prevalence if MVCs were included [10,36] and 16.7%-35.2% if MVCs were excluded [5,6,30]. One study which included cases categorized as “child abuse suspected” in the trauma registry and which received the lowest child abuse methodology rating of 5, reported an abuse prevalence of 68.3% in children <18 months old [27]. In a study of children ≤36 months old with a reported stair fall history, 13.8% of children with femur fractures were categorized as abused [31]. Among children >12 months old with all types of femur fractures, abuse was diagnosed or confirmed in 1.5-6.0% of cases [10,18,30].

### Probability of abuse diagnosis in young children with femur fractures: diaphyseal type

**Figure 3:**
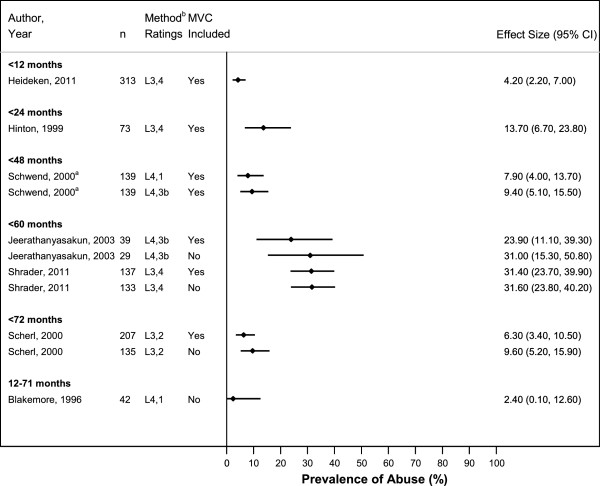
**Probability of Abuse in Children with Diaphyseal Femur Fractures. **^a^Study was limited to children with isolated femur fractures without additional injuries. ^b^Presents overall study methodology ranking (L1-L5) and abuse determination methodology ranking (1–5).

In a single Swedish study relying on administrative data, 4.2% of children <12 months old with diaphyseal femur fractures were categorized as abused (Figure 3) [34]. A higher abuse prevalence, 13.7%, was reported in an American study of children <24 months old, also relying on administrative data [23]. Two studies, one American and one Thai, reported even higher rates of suspected abuse among children <60 months old with non-MVC-related diaphyseal femur fractures: 31.6% and 31.0% respectively [32,33]. Two studies focused on children with diaphyseal femur fractures and no additional injuries reported lower prevalences of cases substantiated as abusive by Child Protective Services (CPS) in their samples (2.4% and 7.9%) [22,26].

### Probability of abuse diagnosis in young children with femur fractures: distal metaphyseal type

In the single study of children <12 months old with a complete distal metaphyseal fracture, abuse was suspected in 75% and diagnosed in 50% of cases [28].

### Patient and family demographics associated with abuse determination

Age <12 months was associated with increased abuse likelihood in 3 of 5 studies, with positive likelihood ratios ranging from 3.3-19.7 (Table 4) [10,20,24,30,32]. Another study reported increased probability of abuse among children aged <18 months, as compared to counterparts ≥18 months [2]. Two studies examined the relationship between age in years as a continuous variable and risk of abuse: one found significant association between younger age and increased risk [26], but the other did not [22]. Non-ambulatory status was associated with increased abuse likelihood in both studies examining this association [26,30].

**Table 4:** Association of demographic characteristics with likelihood of abuse

**Characteristics**	**Study**	**Sensitivity (95% CI) (95%CI)**	**Specificity (95%CI)**	**LR+ (95% CI)**	**LR- (95% CI)**	***P***^**a**^
**Age**						
< 12 m.o. vs. 12 m.o.-35 m.o.	Thomas, 1991	66.7 (35.9-97.5)	75.0 (53.8-96.2)	2.7 (1.0-7.0)	0.4 (0.2-1.2)	0.09
< 12 m.o. vs. 12 m.o.-35 m.o.	Hui, 2008	71.4 (47.8-95.1)	55.8 (46.6-64.9)	1.6 (1.1-2.4)	0.5 (0.2-1.2)	0.09^b^
< 12 m.o. vs. 12 m.o.-35 m.o.	Leventhal, 2008	81.6 (78.0-85.0)	75.4 (74.0-76.8)	3.3 (3.1-3.6)	0.2 (0.2-0.3)	**<0.001**
< 12 m.o. vs. 12 m.o.-59 m.o.	Rex, 2000	92.9 (79.4-100.0)	73.7 (53.9.-93.5)	3.5 (1.6-7.6)	0.1 (0.0-0.7)	**<0.001**
< 12 m.o. vs. 12 m.o.-71 m.o.	Shrader, 2011	41.9 (27.1-56.6)	97.9 (95.0-100.0)	19.7 (4.8-81)	0.6 (0.5-0.8)	**<0.001**
< 18 m.o. vs. 18 m.o.-47 m.o.	Baldwin, 2011	90.0 (83.0-97.0)	68.3 (60.6-76.1)	2.8 (2.2-3.7)	0.1 (0.1-0.3)	**<0.001**
**Ambulatory Status**						
Non-ambulatory vs. ambulatory	Schwend, 2000	76.9 (54.0-99.8)	88.1 (82.4-93.7)	6.5 (3.7-11.3)	0.3 (0.1-0.7)	**<0.001**
Non-ambulatory vs. ambulatory	Hui, 2008	71.4 (47.8-95.1)	69.0 (60.5-77.6)	2.3 (1.5-3.5)	0.4 (0.2-1.0)	**0.006**
**Health Insurance Status**						
Uninsured vs. insured	Baldwin, 2011	7.1 (1.2-13.2)	90.6 (85.8-95.5)	0.8 (0.3-2.0)	1.0 (0.9-1.1)	0.80
Uninsured/Medicaid vs. private	Shrader, 2011	76.7 (64.1-89.4)	51.1 (41.0-61.2)	1.6 (1.2-2.0)	0.5 (0.3-0.8)	**0.003**
Uninsured/Medicaid vs. private	Blakemore, 1996	37.5 (13.8-61.2)	88.5 (76.2-100.0)	3.3 (0.9-11.2)	0.7 (0.5-1.1)	0.06
**Gender**						
Male vs. female	Schwend, 2000	61.5 (35.1-88.0)	27.8 (20.0-35.6)	0.9 (0.5-1.3)	1.4 (0.7-2.9)	0.52
Male vs. female	Baldwin, 2011	51.4 (39.7-63.1)	32.4 (24.6-40.2)	0.8 (0.6-1.0)	1.5 (1.1-2.1)	**0.002**
Male vs. female	Hui, 2008	50.0 (23.8-76.2)	37.2 (28.3-46.1)	1.2 (0.7-2.0)	0.8 (0.5-1.4)	0.39
Male vs. female	Rewers, 2005	62.5 (48.4-76.2)	31.7 (26.2-37.1)	0.9 (0.7-1.2)	1.2 (0.8-1.8)	0.51
Male vs. female	Arkader, 2007	80.0 (55.2-100)	40.0 (9.6-70.4)	1.3 (0.7-2.4)	0.5 (0.1-2.1)	0.63
**Race**						
Black vs. White/other	Schwend, 2000	46.2 (19.1-73.3)	77.0 (69.6-84.3)	2.0 (1.0-3.9)	0.7 (0.4-1.2)	0.09
Black vs. White/Hispanic/other	Rewers, 2005	54.2 (40.1-68.3)	44.2 (38.4-50.1)	1.0 (0.7-1.3)	1.0 (0.7-1.4)	0.88

^a^Computed using two-sided Fisher’s exact test. *P*-values of 0.05 or less were considered statistically significant and are presented in bold text.

^b^*P*-value reported in Hui [30] paper was 0.037 but calculated to be 0.09 using two-sided Fisher’s exact test.

Male gender and Medicaid/uninsured status were each associated with increased likelihood of abuse likelihood in only one [2,32] of several studies examining these associations [22,26,28,30,35]. No association was found between abuse likelihood and race [26,35]. In a single study, lower scores on the Hollingshead Occupational Scale (HOS) for fathers, but not mothers, were associated with reports to CPS [22].

### History characteristics associated with abuse

A suspicious history was associated with abusive femur fracture in 3 of 3 studies (Table 5) [2,22,30]. The definition of suspicious history varied but generally included history of no trauma, an unwitnessed trauma history, or a history considered inconsistent with the injury. Delay in care of >24 hours and unknown trauma mechanism were associated with abuse, while a trauma history witnessed by a non-parent was associated with non-abusive femur fracture [22,30,32]. In one study, injuries occurring at home or an unknown location were associated with abuse compared to injuries occurring in public places [35].

**Table 5:** A**ssociation of History & Examination Characteristics with Likelihood of Abuse**

**Characteristics**	**Study**	**Sensitivity (95%CI)**	**Specificity (95%CI)**	**LR+ (95% CI)**	**LR- (95% CI)**	***p***^**a**^
**Reported History of Trauma**^**c**^						
Suspicious vs. non-suspicious	Blakemore, 1996	68.7 (46.0-91.5)	88.0 (75.3-100.0)	5.7 (1.9-17.4)	0.4 (0.2-0.7)	**<0.001**
Suspicious vs. non-suspicious	Baldwin, 2011	32.9 (21.9-43.9)	95.7 (92.3-99.1)	7.6 (3.2-17.8)	0.7 (0.6-0.8)	**<0.001**
Suspicious vs. non-suspicious	Hui, 2008	71.4 (47.8-95.1)	97.3 (94.4-100.0)	26.9 (8.4-86.2)	0.3 (0.1-0.7)	**<0.001**
Unknown vs. known history	Shrader, 2011	39.5 (24.9-54.1)	94.7 (90.1-99.2)	7.4 (2.9-18.8)	0.6 (0.5-0.8)	**<0.001**
Unwitnessed vs. witnessed	Blakemore, 1996	43.8 (19.4-68.1)	72.0 (54.4 -89.6)	1.6 (0.7-3.6)	0.8 (0.5-1.3)	0.33
Witnessed by non-parent (yes vs. no)	Blakemore, 1996	18.8 (0.0-37.9)	48.0 (28.4-67.6)	0.4 (0.1-1.1)	1.7 (1.1-2.7)	**0.05**
History of fall vs. other history	Blakemore, 1996	93.8 (81.9-100.0)	26.9 (9.9-44.0)	1.3(1.0-1.7)	0.2 (0.0-1.7)	0.13
**Reported time from injury to care**						
Delay >24 hours vs. no delay	Hui, 2008	42.9 (16.9-68.8)	92.0 (87.0-97.0)	5.4 (2.3-12.9)	0.6 (0.4-1.0)	**0.002**
**Location Injury Occurred**						
Home/unknown vs. public place^d^	Rewers, 2005	97.9 (93.9-100)	22.5 (17.65-27.4)	1.2-1.4	0.1 (0.0-0.7)	**<0.001**
**Additional Injuries**						
Fractures, bruises, or SDHs (yes vs. no)	Hui, 2008	42.9 (16.9-68.8)	92.0 (87.0-97.0)	3.7 (1.7-8.2)	0.6 (0.4-1.0)	**0.007**
Bruises vs. no bruises	Pierce, 2005	100 (NA^e^)	72.0 (NA^e^)	3.6 (1.9-6.7)	0.0 (NA)	**0.01**^**b**^
Current polytrauma^e^ (yes vs. no)	Baldwin, 2011	52.9 (41.2-64.6)	92.1 (87.6-96.6)	6.7 (3.6-12.3)	0.5 (0.4-0.7)	**<0.001**
Occult injury on imaging (yes vs. no)	Pierce, 2005	75.0 (19.4-99.4)	100.0 (86.0-100.0)	NA	0.3(0.0-1.4)	**0.001**
Any	Shrader, 2011	20.9 (8.8-33.1)	90.4 (84.5-96.4)	2.2 (0.9-5.1)	0.9 (0.7-1.0)	0.10

^a^Computed using two-sided Fisher’s exact test. *P-*values of 0.05 or less were considered statistically significant and are presented in bold text.

^b^*P*-value reported in Pierce [31] was 0.055 but computed as 0.010 using two-sided Fisher’s exact test.

^c^Definition of suspicious history generally included no known history of trauma, or a history of trauma that was unwitnessed or considered inconsistent with the injury.

^d^Public place included locations categorized as public, recreation, and street.

^e^Current polytrauma was defined as another concurrent long bone, clavicle, axial fracture, or other body system injury that would require hospitalization.

### Additional injuries associated with abuse

Additional injuries, including bruises, other fractures identified on skeletal survey, and subdural hemorrhages, were significantly associated with determination of abusive femur fracture in 3 of 4 studies (Table 5**)**[2,30-32]. Universal screening for additional injuries was, however, not performed in any of these studies.

### Fracture characteristics associated with abuse

In 2 of 3 studies, diaphyseal fractures were associated with decreased abuse likelihood, compared to other fracture types (Table 6) [2,30,35]. Distal diaphyseal fractures, in comparison with proximal and midshaft diaphyseal fractures, were associated with abuse in another study [26]. In one report, distal metaphyseal fractures showed increased abuse probability [2], but this finding was not replicated in a second study [30]. Fracture side, spiral fracture type, or bilateral fractures did not show significant association with abuse [22,26,28,30].

**Table 6:** Association of fracture characteristics with likelihood of abuse

**Characteristics**	**Study**	**Sensitivity (95%CI)**	**Specificity (95%CI)**	**LR+ (95% CI)**	**LR- (95% CI)**	***p***^**a**^
**General Fracture Position**						
Diaphyseal vs. all other	Hui, 2008	78.6 (57.1-100.0)	36.3 (27.4-45.1)	1.2 (0.9-1.7)	0.6 (0.2-1.7)	0.38
Diaphyseal vs. all other	Rewers, 2005	58.3 (44.4-72.3)	22.4 (17.5-27.3)	0.8 (0.6-1.0)	1.9 (1.2-2.8)	**0.007**
Diaphyseal vs. all other	Baldwin, 2011	44.4 (33.0-55.9)	33.8 (25.9-41.7)	0.7 (0.5-0.9)	1.6 (1.2-2.2)	**0.003**
Subtrochanteric vs. all other	Hui, 2008	7.1 ( 0.0-20.6)	95.6 (91.8-99.4)	0.5 (0.1-1.8)	1.2 (0.9-1.5)	0.51
Subtrochanteric vs. all other	Baldwin, 2011	19.4 (10.3-28.6)	86.3 (80.6-92.0)	1.4 (0.8-2.7)	0.9 (0.8-1.1)	0.32
Distal metaphyseal vs. all other	Hui, 2008	14.3 (0.0-32.6)	70.8 (62.4-79.2)	0.5 (0.1-1.8)	1.2 (0.9-1.5)	0.35
Distal metaphyseal vs. all other	Baldwin, 2011	36.1 (25.0-47.2)	79.9 (73.2-86.5)	1.8 (1.1-2.8)	0.8 (0.7-1.0)	**0.02**
**Diaphyseal Fracture Position**						
Distal vs. mid/proximal	Schwend, 2000	53.8 (26.7-80.9)	88.4 (82.7-94.1)	4.7 (2.3-9.4)	0.5 (0.3-0.9)	**0.001**
**Diaphyseal Fracture Type**						
Spiral vs. non-spiral	Blakemore, 1996 11119961996	68.8 (46.0-91.5)	11.5 (0.0-23.8)	0.8 (0.5-1.1)	2.7 (0.7-9.8)	0.23
Transverse vs. all other	Pierce, 2005	75.0 (32.6-100.0)	84.0 (69.6-98.4)	4.7 (1.6-13.6)	0.3 (0.1-1.6)	0.03
**Fracture Side**						
Left vs. right	Schwend, 2000	58.3 (30.4-86.2)	39.5 (30.9-48.1)	1.0 (0.6-1.6)	1.1 (0.5-2.1)	1.00
Left vs. right	Blakemore, 1996	37.5 (13.8-61.2)	46.2 (27.0-65.3)	0.7 (0.3-1.4)	1.4 (0.8-2.4)	0.35
Left vs. right	Hui, 2008	57.1 (31.2-83.1)	52.2 (43.0-61.4)	1.2 (0.7-2.0)	0.8 (0.4-1.5)	0.58
Left vs. right	Arkader, 2007	60.0 (29.6-90.4)	30.0 (1.6-58.4)	0.9 (0.4-1.6)	1.3 (0.4-4.5)	1.00
**Bilateral**						
Bilateral vs. unilateral	Schwend, 2000	7.7 (0.0-22.2)	98.4 (96.2-100.0)	4.8 (0.5-49.9)	0.9 (0.8-1.1)	0.26

^a^Computed using two-sided Fisher’s exact test. *P*-values of 0.05 or less were considered statistically significant and are presented in bold text.

## Discussion

Our review identified 24 studies providing information about prevalence of abuse among young children with femur fractures. The confirmed or suspected abuse prevalence varied widely across the studies with point estimates ranging from 1.5% - 68.3%, reflecting the heterogeneity of the studies’ populations and methodologies. The structure of this review, focusing on smaller groups of studies with similar populations and stricter child abuse determination methods, produced narrower ranges of abuse proportions.

Our results indicate that there is substantial risk of abuse (16.7%- 35.5%) in children <12 months old with non-MVC-related femur fractures. The abuse prevalence in this age group was lower, however, than the 50.1% (95% CI, 34.1-66.1) reported for children <18 months in a recent meta-analysis by Maguire et al., [8] likely due to differences in search strategy and inclusion criteria. We included accidental trauma as well as abusive trauma search terms to prevent bias towards studies focused on abusive fractures alone. In addition, we limited our search to articles published in 1990 or later, while Maguire et al. included earlier papers. Although the shorter time frame for our review may have excluded relevant papers, we included only papers published after 1990 for two important reasons. First, data suggest that the abuse probability among children with fractures has changed over time [29]. Second, the subject matter is explored mostly in retrospective studies which depend upon clinical evaluation practices that may have changed over time. Child abuse is a relatively new field; many pediatric centers did not establish child abuse programs until after 1990 [37]. Finally, our estimates for prevalence of abusive femur fractures included data from cohort and cross-sectional studies, while Maguire et al. also extrapolated from case–control studies. Although the two reviews provide different point estimates, both found a high prevalence of abuse in children <12 months old with femur fractures.

The results of this review also emphasize the significance of reported history and the presence of additional injuries for abuse determination in young children with femur fractures. Femur fracture type and position were, in general, not significantly associated with abuse, with the exception that diaphyseal fractures and mid/proximal diaphyseal fractures particularly might be associated with decreased abuse likelihood. On the other hand, distal metaphyseal femur fractures may indicate increased likelihood of abuse.

This review has three principal limitations. First, the studies were mostly retrospective in design and not all subjects were assessed consistently for clinical findings, potentially contributing to detection bias. Second, as there is no clear gold standard for diagnosing child abuse, there is concern for circular reasoning in the identification of factors associated with increased risk of abuse. Third, influence of the abuse determination method on abuse prevalence estimates was evident: studies employing more stringent criteria for making a determination of abuse reported lower abuse rates than studies with less stringent criteria. This observation is highlighted in a study where 38% of cases were reported to CPS for suspected abuse, but only 2% were confirmed secondary to abuse at court hearings [22]. Another key concern lies in the potential differences across physicians in calibration of their determinations of abuse [38,39]. As a result, the variability of findings of abuse might reflect the spectrum of criteria for those determinations, rather than an underlying variation in the prevalence of abuse if all studies applied the same criteria for determining abusive fractures.

The results of this review underscore the need for prospective studies of children with femur fractures to allow for more accurate estimation of abuse risk in this population. In such studies, standardized evaluation and data collection procedures would be utilized to minimize detection bias and circular reasoning. Lacking a gold standard method for determining abuse, these ideal studies would report whether abuse was diagnosed using specific stated case criteria, as well as whether abuse was confirmed by CPS or court proceedings.

## Conclusions

This comprehensive literature review underscores the high prevalence of abuse among children <12 months old with femur fractures. In addition, non-ambulatory status, a suspicious history, and the presence of additional injuries were associated with increased likelihood of abusive femur fracture in multiple studies. No significant association was found between probability of determination of abuse and the following characteristics: fracture side; spiral fracture type; bilateral fractures. Additional prospective studies are needed to further elucidate the characteristics that affect abuse probability in children with femur fractures.

## Abbreviations

cps: Child protective services; mvc: Motor vehicle crash.

## Competing interests

The authors do not have conflicts of interest to disclose.

## Authors’ contributions

JW conceived of the systematic review and participated in design of the review, development of the search strategy, screening and assessment of articles, data abstraction, statistical analysis and manuscript drafting. OF participated in design of the review, development of the search strategy, screening and assessment of articles, data abstraction, statistical analysis, and manuscript drafting. VM participated in design of the review, screening and assessment of articles and manuscript editing. RL participated in design of the review, statistical analysis and manuscript editing. CF and DR participated in design of the review and manuscript editing. All authors read and approved the final manuscript.

## Pre-publication history

The pre-publication history for this paper can be accessed here:

http://www.biomedcentral.com/1471-2431/14/169/prepub

## Supplementary Material

Additional file 1 **Search Terms.** Terms used for performing searches in the PubMed/MEDLINE database. Studies with a publication type of “case report” were excluded. An almost identical set of terms was used for searches in the CINAHL database. Click here for file

Additional file 2 **PRISMA Checklist. ***From:* Moher D, Liberati A, Tetzlaff J, Altman DG, The PRISMA Group (2009). Preferred Reporting Items for Systematic Reviews and Meta-Analyses: The PRISMA Statement. PLoS Med 6(6): e1000097. Click here for file
