# Supplementary material for: Prevalence of abuse among young children with femur fractures: a systematic review
Source: BMC Pediatr. 2014 Jul 2;14:169. doi: 10.1186/1471-2431-14-169 (PMC4085378; doi:10.1186/1471-2431-14-169)
Supplement: Additional file 1 — Search Terms. Terms used for performing searches in the PubMed/MEDLINE database. Studies with a publication type of “case report” were excluded. An almost identical set of terms was used for searches in the CINAHL database. [file 1471-2431-14-169-S1.docx]

| **Search** | **Terms** | **# of articles** |
| --- | --- | --- |
| 1 | child abuse [MeSH Terms]) | 14,598 |
| 2 | shaken baby syndrome [MeSH Terms] | 284 |
| 3 | battered child syndrome[MeSH Terms] | 148 |
| 4 | 1 or 2 or 3 | 14,759 |
| 5 | child abuse [Title/Abstract] or child maltreatment [Title/Abstract] or battered child [Title/Abstract] | 4,508 |
| 6 | 4 or 5 | 15,818 |
| 7 | physical abuse [Title/Abstract] | 2,941 |
| 8 | multiple trauma [MeSH Terms] | 4,218 |
| 9 | accidents [MeSH Terms] | 75,387 |
| 10 | trauma*[Title/Abstract] | 122,939 |
| 11 | injur*[Title/Abstract]) | 334,995 |
| 12 | accident*[Title/Abstract} | 35,939 |
| 13 | 7 or 8 or 9 or 10 or 11 or 12 | 480,442 |
| 14 | pediatrics [MeSH Terms] or child"[MeSH Terms] or infant"[MeSH Terms] | 813,211 |
| 15 | child*[Title] or infant*[Title/Abstract] or pediatric*[Title/Abstract] | 653,084 |
| 16 | 14 or 15 | 998,950 |
| 17 | 13 and 16 | 64,327 |
| 18 | fractures, bone"[MeSH Terms]2 | 50,515 |
| 19 | fracture*[Title/Abstract] | 85,251 |
| 20 | 18 or 19 | 934,345 |
| 21 | 17 and 20 | 6,239 |
| 22 | 6 and 20 | 469 |
| 23 | 21 or 22 | 6,385 |
